# Novel gene and gene model detection using a whole genome open reading frame analysis in proteomics

**DOI:** 10.1186/gb-2006-7-4-r35

**Published:** 2006-04-28

**Authors:** Damian Fermin, Baxter B Allen, Thomas W Blackwell, Rajasree Menon, Marcin Adamski, Yin Xu, Peter Ulintz, Gilbert S Omenn, David J States

**Affiliations:** 1 Bioinformatics Program, University of Michigan, Ann Arbor, MI 48109, USA; 2 Department of Internal Medicine, University of Michigan, Ann Arbor, MI 48109, USA; 3 Department of Human Genetics, University of Michigan, Ann Arbor, MI 48109, USA

## Abstract

**Background:**

Defining the location of genes and the precise nature of gene products remains a fundamental challenge in genome annotation. Interrogating tandem mass spectrometry data using genomic sequence provides an unbiased method to identify novel translation products. A six-frame translation of the entire human genome was used as the query database to search for novel blood proteins in the data from the Human Proteome Organization Plasma Proteome Project. Because this target database is orders of magnitude larger than the databases traditionally employed in tandem mass spectra analysis, careful attention to significance testing is required. Confidence of identification is assessed using our previously described Poisson statistic, which estimates the significance of multi-peptide identifications incorporating the length of the matching sequence, number of spectra searched and size of the target sequence database.

**Results:**

Applying a false discovery rate threshold of 0.05, we identified 282 significant open reading frames, each containing two or more peptide matches. There were 627 novel peptides associated with these open reading frames that mapped to a unique genomic coordinate placed within the start/stop points of previously annotated genes. These peptides matched 1,110 distinct tandem MS spectra. Peptides fell into four categories based upon where their genomic coordinates placed them relative to annotated exons within the parent gene.

**Conclusion:**

This work provides evidence for novel alternative splice variants in many previously annotated genes. These findings suggest that annotation of the genome is not yet complete and that proteomics has the potential to further add to our understanding of gene structures.

## Abstract 2

High-throughput mass spectroscopy data combined with a six-frame translation of the human genome can be used to identify novel protein encoding genes, as demonstrated with a search for plasma proteins.

## Background

Defining the location of genes and the precise nature of gene products remains a fundamental challenge in genomics. High throughput tandem mass spectrometry based proteomics provides an important new source of information to help define both the location of transcription units and the reading frame of protein translation. In theory, high throughput proteomics will complement genome and transcript sequence analysis by independently confirming translation products. In practice, a number of technical challenges have limited the widespread use of this approach. In this paper, we present a novel statistical approach to assessing the significance of peptide and open reading frame (ORF) matches when searching very large target sequence collections. We further demonstrate that these measures allow us to identify a substantial number of new gene models by comparing the tandem mass spectra data of the Human Proteome Organization (HUPO) Plasma Proteome Project (PPP) against the amino acid sequences coded by all of the ORFs in the human genome. The use of an exhaustive translation of the human genome also allows us to identify many peptides not contained in the standard protein sequence collections.

In the five years since the first draft of the human genome was released, it has undergone numerous revisions primarily in the form of additional gene annotations. However, despite the fact that we live in a post-genomic era, there is still much to be learned from the sequence that is the basic blueprint for humans. As the number of genome entries in public databases has expanded in recent years, *de novo *gene prediction has been greatly improved. New approaches have been developed that employ multiple genome alignments to make better gene predictions [1-3]. Along with these new gene predictors, empirical data from expressed sequence tags (ESTs) are also being exploited in the search for novel coding regions [4,5]. Despite these advances, there still remains a great deal of uncertainty regarding the current gene model [6].

High throughput, bottom up chromatography/tandem mass spectrometry protein identification strategies, makes possible a new approach to human genome annotation: identifying all known proteins. Using mass spectrometry (MS) data, it is now possible to work backwards from a protein to its parent genomic sequence. Previous work has been done using mass spectra for *de novo *gene finding [7]. Recently, Desiere *et al *. [8] performed such an analysis using their MS data. In their work they were able to map 25,754 of their 26,840 peptides to 9,747 of the human Ensembl proteins. Kuster *et al *. [9] and Choudhary *et al *. [10] both used the draft sequence of the human genome as a template to search for novel peptides.

One of the major limitations of protein identification by MS is that all current software packages rely on a protein database against which to search. As a result, even the most exhaustive protein database search is limited to the data available in the current public databases. This poses a serious constraint if one is searching for novel protein coding regions since all results will be limited to data for a small set of highly curated proteins. In this paper, we describe an exhaustive protein database generated from the 6-frame translation of the entire human genome to identify peptides isolated from human blood. Peptides found from the MS data of the Human PPP were mapped back to their parent sequences using this database [11]. Our method revealed a number of splice variants to previously annotated genes as well as several new coding regions that potentially encode novel exons. These candidate regions were validated using EST mapping.

## Results

### Identifying novel splice variants

Since our goal was to identify novel coding regions including splice variants, we needed to obtain all the possible ORFs encoded by the genome. To this end we generated a putative open reading frame FASTA file for each chromosome. These ORF sequences were obtained by translating each chromosome in all six reading frames. This method of generating a putative ORF library did not take into account global genomic features such as exon/intron splice boundaries or repeat regions. Therefore, the method produced a significant number of protein sequences that were unlikely to be real. The average length of a sequence in our library of ORFs is 25.5 residues (± 22.6 standard deviations). In contrast, the average protein length of an entry in the International Protein Index (IPI) database (release 3.14) is 438.5 amino acids (± 523.8 standard deviations) [12]. This suggests an overabundance of relatively short peptide sequences in our protein data set. Our method, however, ensured that we obtained a representative for every possible exon encoded in the human genome. We were willing, therefore, to accept this initial high degree of signal to noise in our putative ORF library.

### Selection of diagnostic peptides

**Figure 1:**
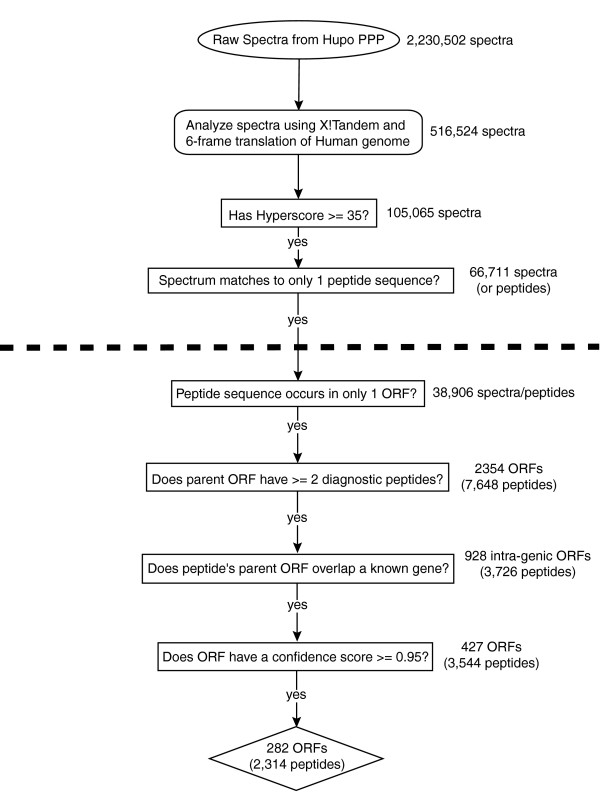
Selection of candidate high confidence ORFs. The flowchart diagrams how high confidence ORFs were identified. Data starts with raw spectra being analyzed by X!Tandem using our six-frame genome translation and ends with our set of high confidence ORFs and the peptides contained within them. The dashed line indicates the switch from discussion of spectra/peptides to ORFs.

The ORF library was used as the search database for X!Tandem, an open source program that matches tandem mass spectra to a peptide sequence from a given database of protein sequences [13,14]. As mentioned earlier, the putative ORF library used by X!Tandem contained a very high degree of noise. As a result, the peptide identifications resulting from this analysis needed to be filtered to remove false hits. As an initial filtering step, spectra whose X!Tandem peptide matches had hyperscores below 35 were removed from consideration. Large hyperscore values indicate that the match made by X!Tandem was a high confidence one. This threshold was chosen based upon analysis of a hyperscore receiver-operator curve(ROC) generated from a collection of known high confidence matches and a set of known false negatives (see Materials and methods). In choosing a threshold of 35, we reduced the number of potential false positive matches made by X!Tandem. This reduced our search space to one-fifth its original size (516,524 spectra reduced to 105,065 spectra). Many spectra were matched to multiple peptide sequences. In these instances, it would be difficult to determine which peptide is the true match to the spectra. To avoid this ambiguity, we selected for spectra that were only matched to a single unique peptide sequence. From these peptides, we selected out only those that were unique to a single ORF in the database. This left us with 38,906 peptide matches that we are considering our set of high-confidence diagnostic peptides. In the flowchart presented in Figure 1, this corresponds to the first box below the dashed line.

### Selection of candidate open reading frames

To identify potential novel coding regions, the diagnostic peptides were mapped back to their parent ORFs. A total of 33,502 putative ORFs contained at least 1 diagnostic peptide. High confidence ORFs as identified using our Poisson probability (see Materials and methods), which had at least 2 diagnostic peptide matches, were selected. Based on these criteria, we isolated a total of 427 ORFs that were represented by 3,544 diagnostic peptides. Candidate ORFs were then separated into two major categories based upon whether or not their coordinates overlapped with those of an annotated gene. A total of 282 ORFs (represented by 2,314 peptides) were classified as intragenic ORFs. The information presented up to this point is contained within the flowchart of Figure 1. We then analyzed these ORFs and their associated peptides in greater detail.

### Analysis of intragenic open reading frame peptides

**Figure 2:**
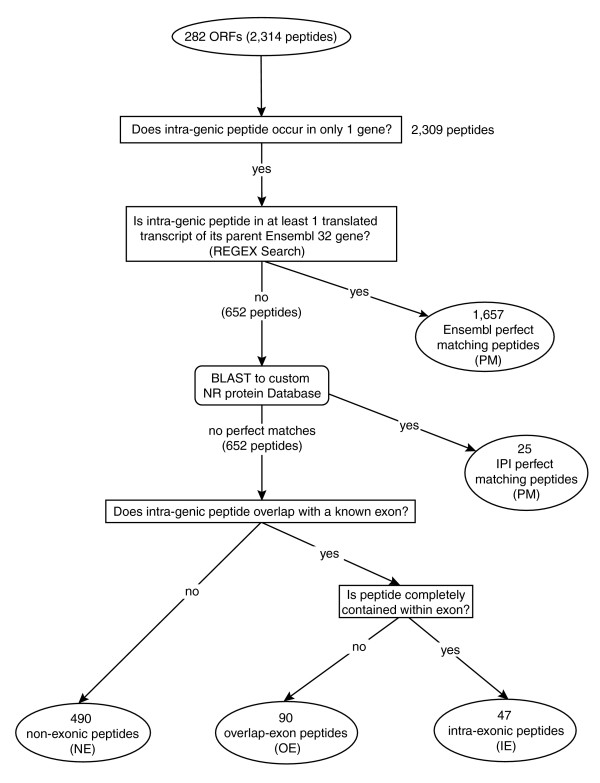
Selection and classification of diagnostic peptides. The flowchart outlines how diagnostic peptides found in high-confidence ORFs were classified into four categories: perfect match (PM), intra-exonic (IE), overlapping exon (OE), and non-exonic (NE).

To validate our method, we examined the peptides derived from intragenic ORFs in terms of how well they matched to known protein coding regions. Work from this section is illustrated in the flowchart in Figure 2. Of the 2,314 intragenic peptides, 5 were derived from an ORF that straddled 2 different gene coding regions. Since we were unable to determine which gene produced which peptide, all five were discarded. The remaining 2,309 were unique to a single gene and their peptide sequences were searched against a non-redundant human protein database for exact matches. A total of 1,682 (72.8%) of the intragenic peptides had exact matches to the protein products of the genes they occur within. These peptides were classified as perfect matching (PM) peptides. A total of 89 distinct proteins encompassed all of these PM peptides.

The remaining 627 intragenic peptides do not have a perfect match to a known protein product. This suggests that these peptides represent novel protein products for the genes within which they occur. These peptides were classified into three distinct categories depending upon their position relative to the genomic coordinates of an annotated gene. There were 47 peptides that occurred inside of an annotated exon of their parent gene, but in a different reading frame. These we called (IE) intra-exonic peptides. Another 90 peptides overlapped with a portion of an annotated exon (overlapping exons (OEs)) and the remaining 490 peptides fell in between the coordinates of annotated exons in their parent gene (non-exonic (NE)). Taken together, a total of 128 genes were represented by these intragenic peptides. Table 1 lists the breakdown of all the intragenic peptides. A total of 128 genes encompassed all of our intragenic peptides. Table 2 lists a sampling of the 128 genes along with the peptide breakdown for each gene. A complete list is provided in Additional data file 1.

Of the 128 genes listed, 20 had peptide matches that only occur in non-coding regions. Upon closer inspection of these peptides, most of them contained long repeats of glycine, suggesting that they may be erroneous matches. Rather than discarding these hits immediately, we first tried to determine if there was expression data supporting the observed novel peptide.

### Verification of novel peptides through ESTs

We searched the EST library using our set of diagnostic peptides. If these peptides were in fact being translated, we would expect to identify transcripts encoding them. For this analysis, the DNA sequence encoding the peptide plus 100 base-pair flanking sequence was used in an alignment search. In instances where peptides were substrings of one another, only the longest representative peptide was used as a BLAST query. By doing so, we reduced the number of diagnostic peptides from 2,314 to 1,202. Only hits involving some part of the peptide's coding region were considered true matches. Table 3 gives the breakdown of peptides in the four categories: OE, NE, IE, and PM. The peptide category with the most EST hits was the PM category. Of the peptides that occurred within known exons but in different reading frames, 24 (62%) of them had EST matches, while 36 (65%) of the peptides that overlapped partially with known exons also had EST hits.

Table 4 gives a representative list of the peptide-to-EST matches by gene. The complete list is available in Additional data file 2. A total of 114 genes had diagnostic peptides associated with them that also had EST matches to those peptides. This accounted for 89% of the total genes we reported as having a diagnostic peptide match. The genes having the most EST matches were proteins commonly found in plasma. Only 9 of the 20 genes mentioned earlier for their sole representation by NE peptides had EST supporting evidence for their assigned peptides. Upon inspection of the amino acid sequences for their peptides, it was found that 40 of them (representing 3 genes) were predominately glycine repeats. Overall, a total of 14 identified genes were discarded from the list given in Additional data file 1 since it was more likely that their reported peptide matches were erroneous.

A total of 47 of the 114 genes had EST hits to peptides that were classified as either NE (49 peptides) or OE (52 peptides). These matches potentially represented novel coding regions. The longest conserved block of ESTs that overlapped with a peptide's encoding coordinates were used to better define the boundaries of the novel coding region. Eighty of the 101 novel coding regions (represented by 43 genes) had well defined boundaries that were supported by ESTs. Additional data file 3 summarizes the coordinates for each of the novel OE and NE coding regions found within the 43 genes.

### Protein features of encompassing genes

We examined the annotated protein products of the genes having the novel coding regions defined by OE peptides. NE and IE peptides also represented novel protein products but, with MS data alone, we were unable to accurately define the boundaries for the novel coding region. A new protein product containing the OE peptide sequence was generated and searched against PROSITE and UNIPROT to determine what impact, if any, the addition of the diagnostic peptide fragment would have on the protein's domains. A total of 11 diagnostic peptides overlapped in some way with a known protein domain. Table 5 summarizes the domains identified for the protein products of the genes.

In all cases, the impact caused by the presence of the extra amino acids introduced by the OE peptide was limited to a single domain. PROSITE was able to identify the domain regardless of the presence or absence of the extra amino acid characters, suggesting that the functional components of the domains remained intact and were thus not disrupted by the additional amino acid residues. A review of the literature revealed that all but one of the domains overlapped by the peptides were associated with plasma proteins. The remaining domain is called sirtuin and is reported to function in peptide deacetylation in an NAD-dependent manner. The proteins having this domain are members of the sirtuin family. These proteins are associated with cellular functions involving transcriptional silencing, cell cycle progression, and chromosome stability [15].

## Discussion

We identified a number of novel splice variants to previously annotated genes. These splice variants were identified working backwards from MS data to their parent-coding region in the genome. A six-frame translation of the entire human genome was used as the query database for the protein identification analysis. This enabled us to detect protein products that are currently not in the public databases. We first investigated peptides that could potentially represent novel splice variants of known genes. A total of 2,309 peptides were isolated whose genomic coordinates placed them singularly within the start/stop points of annotated genes. These peptides were grouped into four categories based upon where their genomic coordinates place them within their parent gene. Of these categories, three represent peptides that in some way overlap with a known exon.

The first two categories represented peptides that were completely contained within annotated exons. The first of these were the intra-exon PM peptides. These represented a control group in our study since they should have mapped to previously annotated proteins. Of our 2,309 high quality peptides, 1,682 (72.3%) fall into this category. The high percentage of peptides in this category that were successfully matched suggested that our methods were sound. The second intra-exonic peptide category consisted of 47 peptides whose coding region was contained within a known exon but whose amino acid sequence corresponded to a different reading frame. The final exonic peptide category was for peptides whose coding regions overlapped partially with those of a known exon. A total of 90 peptides were identified that extended the start or end boundaries for known exons. Apart from the intragenic peptides in the preceding 3 categories, an additional 490 peptides aligned to non-coding regions within genes. These peptides potentially represented novel exons for parent genes that have not been previously identified. This suggests that many genes have splice variants that have not previously been identified. Several reasons for this can be put forward, including sequencing errors and polymorphisms. Both of these may result in frame shift mutations that could prematurely end a coding exon or extend an intron. It is also possible that these ORFs were overlooked because they did not conform to accepted gene models. Many gene prediction algorithms use training data from known coding sequences to identify putative gene regions. Hence, prediction programs may overlook ORFs not fitting their training model. Another possibility is that these ORFs overlap with repeat-rich or low-complexity DNA regions; many sequence analysis tools mask regions that are high in repeats, resulting in these ORFs escaping detection. An additional explanation is that human errors were introduced into the database annotations. These errors, like the frame shift mutations or polymorphisms, would alter the exon/intron splice boundaries.

None of the final 114 genes having peptide matches were annotated as pseudogenes in the ENSEMBL, UCSC or NCBI genome web sites. It is possible that a spectrum could match to an ORF derived from a pseudogene. For relatively recent pseudogenes and processed pseudogenes, the peptide would also match to the true gene from which the unused copy arose. Our filtering methods would eliminate early on such a peptide match. In cases of older and more highly diverged pseudogenes, there might be little to distinguish them from random intergenic sequence. False matches in the database search phase of our algorithm could occur in these regions, but there is no reason to anticipate that they would occur more frequently than false matches in other regions of the genome. The 2,309 intragenic peptides all mapped to 128 distinct genes. Table 2 lists the names of the various proteins encoded by these genes. In looking at the table, it is clear that the vast majority of these proteins are plasma proteins. This is to be expected given that the source of our peak list extractions was human blood plasma. In this study, we used MS data provided by the HUPO PPP consortium. Since these raw data were derived from human plasma, our data were most descriptive for that tissue type as supported by the genes identified. Our approach could easily be applied to other tissue samples. Such an experiment could reveal novel splice variants of other proteins whose expression was unique to the chosen tissue type.

## Conclusion

In this paper, we present a novel approach to assessing the significance of peptide and ORF matches when searching very large target sequence collections. We further demonstrate that these measures allow us to identify a substantial number of new gene models through comparison using tandem mass spectra against the amino acid sequences coded by all of the ORFs in the human genome. We found a large number of genes (114) have either incomplete descriptions of their annotated exons, or potentially novel coding regions. Working backwards from MS data we were able to show supporting evidence for the existence of novel coding regions in previously annotated genes. Most (89%) of the genes we identified as having peptide matches are supported by expression data.

Our use of an exhaustive translation of the human genome has clearly suggested that many genes contain variable splice sites that have not been previously characterized. While this work focused on novel splice variants, the approach could also be used to identify candidate novel ORFs that do not overlap with previously annotated genes. Such ORFs could represent novel genes whose cellular functions have not yet been characterized. Future work will focus on identifying such candidate ORFs and investigating their viability as possible novel genes. Given the extensive literature describing plasma proteins and the stringent statistical requirements applied here, which limit the sensitivity for detecting less abundant species, it is not surprising that we did not find convincing examples of novel genes in this study. Furthermore, this work demonstrates that we can use proteomics to further improve our annotation of the human genome, and it shows that the annotation of the genome is still a work in progress.

## Materials and methods

### Generating the open reading frame database

The complete human genome (NCBI 35 hg17) was downloaded from the UCSC Genome site in FASTA format [16]. Putative ORFs were generated by translating each chromosome starting from its first nucleotide. ORFs were terminated whenever a stop codon was encountered. The next ORF was started at the next nucleotide following the previous stop codon. Instances of ambiguous nucleotides (represented by 'N' in the genome sequence) were replaced with random nucleotides; other ambiguous characters were also replaced with random nucleotides depending upon their symbol. Putative ORFs were generated on both DNA strands of the chromosome in all three reading frames.

The genomic coordinates and orientation were recorded for every novel ORF. Only the first instance of every putative ORF encountered on a chromosome was recorded. Resulting amino acid sequences for each chromosome were recorded in a FASTA formatted sequence file. A total of 217,305,234 putative ORFs were generated using this method. The sequences for these ORFs, along with the source code for the program that generated them, are available for public download at [17].

### Protein identification using X!Tandem

MS data collected as part of the HUPO PPP was used in this study. Briefly, the samples collected were pooled plasma and serum from Caucasian, African and Asian American donors. These data consist of 2,230,502 tandem mass spectrometry (MS/MS) spectra generated by a number of contributing laboratories. Peaklists were either obtained as collections of individual *.dta peaklist files from the contributing authors to the HUPO PPP, or extracted directly from contributed *.RAW files using the Spectrum Mill tool. All peaklists corresponding to individual electrospray runs were converted to Mascot Generic Format (MGF) and concatenated together for faster searching. The raw mass spectra used in our study are publicly available at [16,18].

MS data were analyzed using the X!Tandem open source protein identification package [13,14]. Raw data from each mass spectrum run were submitted to X!Tandem along with a FASTA formatted file representing the six-frame translation of one of the Human chromosomes generated as described above. Searches were performed using a mass error tolerance of +/- 2.0 Daltons, allowing for one post-translational modification (57.022 Daltons added to the amino acid cystine). Proteolytic cleavage specificity was turned off for the searches. All X!Tandem runs were performed on a cluster composed of 106 nodes.

X!Tandem analysis XML output was parsed using Perl scripts and stored in an MS SQL server relational database for further analysis. The X!Tandem output data that were recorded included the genomic loci of each peptide, the putative ORF each peptide was found in and the X!Tandem hyperscore associated with the peptide match. Only spectra matches that were associated with a distinct peptide sequence were considered for further analysis; significantly scoring spectra matching multiple ORFs were removed. ORFs containing these diagnostic peptides were selected out as candidate novel ORFs.

### Localization and selection of diagnostic peptides associated with putative ORFs

Coordinates for all known human genes were obtained from Ensembl (Release 32) using BioPerl and the Ensembl API. Genomic coordinates for peptide matches reported by X!Tandem were compared to known human gene coordinates. Peptides localizing within known genes were termed intragenic, and all non-intragenic peptides were disregarded.

**Figure 3:**
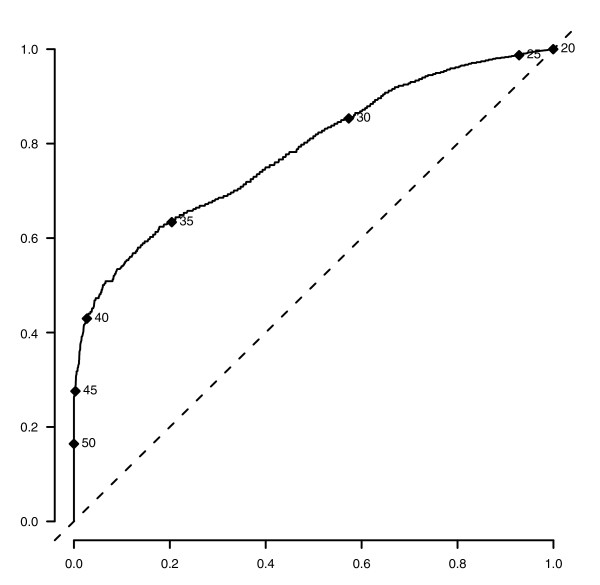
Receiver operator curve for X!Tandem hyperscores. The ROC was used to select the hyperscore cut-off value for candidate peptides. Numbers represent the first instance of the hyperscore values 24, 30, 35, 40, and 45 as they occur among the data points.

We define a diagnostic peptide as one having an X!Tandem hyperscore = 35, mapping to only one genomic locus, and being associated with only one ORF. All peptides meeting these criteria were chosen as diagnostic peptides. The hyperscore threshold of 35 was chosen based upon analysis of a ROC (Figure 3) [19]. Peptides matching to ORFs that were generated from ambiguous nucleotide substitutions were chosen as our set of true negative examples. Spectra matching the 86 most highly represented proteins from the HUPO PPP were used to define our distribution of true positive examples. On the resulting ROC, the first instance of the hyperscore thresholds 25, 30, 35, 40, and 45 were marked.

### Selection of high-confidence putative open reading frames

An important issue in searching very large sequence collections for matches to MS data is assessment of the likelihood of false identification. Several approaches have been utilized [20,21], including probability-based evaluations of mass spectra [8,22,23], reversed sequence database searches [24,25], and Poisson analysis of the identifications by number of peptides matching [26]. However, when applied to the current analysis, these measures exhibited anomalous behavior.

The expected number of peptide matches to a protein depends importantly on the length of the matched protein. We have devised a Poisson model to estimate the expected number of false matches that incorporates the number of spectra searched, score threshold applied for accepting a match, size of the target sequence database and the length of the matched protein sequence. We postulate that a mass spectrum is derived from a given protein in the database but that, in addition, there may be a number of false matches with similar or higher scores occurring at random across the sequence database. Based on the number of proteins for which no peptide matches are reported by any laboratory, we set the rate of matches in our Poisson model, μ, to 1.27197e-5. The mean number of matches, λ, expected at random for a protein of length L is μ*L *. The probability, *P*_*rand *_, that M or more matches will be observed is:



And the expected number of matches, E, is:

*E *= *N*_*db *_*P*_*rand*_

where *N*_*db *_is the number of sequences in the database. The confidence, C, that we have identified the sequence from which the spectral data were derived and not one of the E false positives is:



High-confidence ORFs are defined as those having two or more diagnostic peptide matches and a confidence score of at least 0.95 based upon this Poisson model.

### Identification of diagnostic peptides of known proteins

Intragenic diagnostic peptides mapping to known coding regions were identified using a combination of perfect-match text searching and local sequence alignments. Initial identifications were done by Perl regular expression matching. Peptide sequences were searched against a list of the Ensembl proteins from the genes whose coordinates they overlapped with. Peptides not identified by this method were aligned against the protein products of their parent gene using BLAST, using the PAM30 matrix [27]. In the BLAST searches, only matches of 100% identity were considered. Proteins used in these searches were from a non-redundant set of sequences obtained from the Ensembl genome database (Release 32) [28] and the human IPI database (releases 2.21 and 3.09) [12].

### Classification of novel diagnostic peptides inside of known genes

Intragenic peptides not aligning to a known protein were classified into one of three categories based upon their genomic coordinates in relation to the exons of their parent gene. Novel peptides completely contained within annotated exons were classified as IE. Peptides overlapping annotated exons were classified as overlapping exon OE. Peptides not placed within an annotated exon were classified as NE.

### Alignment of diagnostic peptides against human ESTs

Diagnostic peptides were aligned against the 2005-10-31 release of the human ESTs library obtained from the UCSC genome website [7]. Alignments were performed using MEGABLAST using a word size of 12. For each peptide alignment, the encoding DNA sequence was obtained from the genome flanked by 100 base-pairs. A match was only considered if the EST aligned at least partially to the peptide-encoding region. ESTs aligning to only the flanking regions were discarded.

### Defining novel coding regions

The coding regions, flanked by 1,000 base-pairs, for NE and OE diagnostic peptides were aligned to ESTs. Alignments were performed using BLASTN and only the matches overlapping the peptide coding region and having an E-value less than 1e-6 were accepted. Coordinates for the novel coding region were derived based upon the longest contiguous alignment window generated from overlapping ESTs.

### Identification of disrupted protein domains

Diagnostic peptides classified as OE were aligned to their parent protein using BLASTP. Protein coordinates that would encompass the novel peptide were then computed. Each protein sequence was searched for protein domains using UNIPROT and PROSITE [29,30]. Protein domains overlapped by the novel peptide region were extracted from the database.

Theoretical proteins containing the novel OE peptide sequences were also generated based upon the BLASTP coordinates mentioned above. These theoretical proteins were also analyzed with PROSITE and compared to the original proteins to determine what changes were introduced into the protein domains by the presence of the additional amino acid residues.

### Ensembl DAS viewing of peptides and ORFs

DAS tracks can be viewed by selecting a genomic region using the Ensembl genome browser [31].

## Additional data files

The following additional data are available with the online version of this paper. Additional data file 1 is a Microsoft Excel spreadsheet containing the complete list of 128 genes that have diagnostic peptides associated with them. The table gives the distribution of each type of diagnostic peptide among the 128 parent genes they occur in. The columns in the table are as follows. HUGO Gene ID, HUGO Gene Identifier; Ensembl Gene ID, the Ensembl gene identifier for the gene containing the diagnostic peptides; PT, the total number of diagnostic peptides found within the coding boundaries of this gene; PM, number of perfect-matching peptides to a protein product of this gene; IE, number of intra-exonic peptides associated with this gene; OE, number of exon overlapping peptides associated with this gene; NE, number of non-exonic peptides associated with this gene; Gene Description, a short descriptor characterizing the gene. The gene description is taken from the Ensembl genome browser's record for this gene.

Additional data file 2 is a Microsoft Excel spreadsheet containing the complete list of 114 genes that have diagnostic peptides that are supported by ESTs. The columns of the table are as follows: PT, the total number of non-redundant (NR) peptides associated with this gene; All, the number of peptides with EST hits; PM, number of PM peptides with EST hits; IE, the number of IE peptides with EST hits; OE, the number of OE peptides with EST hits; NE, number of NE peptides with EST hits.

Additional data file 3 is a Microsoft Excel spreadsheet containing the genomic coordinates for the coding region of the novel OE and NE peptides. The table columns are as follows: Peptide Type, indicates whether the anchoring peptide is an NE or OE peptide; Ensembl Gene ID, reports the gene identifier for the gene the peptide occurs in; Chr, Start, and End, report the genomic nucleotide coordinates that encode for the peptide; ESTs, reports how many ESTs overlap with these genomic coordinates.

Additional data file 4 is a Microsoft Excel spreadsheet providing the sequences for the diagnostic peptides and the ORFs they align to. The columns in this spreadsheet are as follows: Ensembl Gene ID, the Ensembl gene identifier for the gene that the ORF overlaps; orflocid, a unique identifier for the ORF sequence; peplocid, a unique identifier for the peptide sequence identified as mapping to this ORF; groupId, an X!Tandem identifier for the spectrum assigned to the given peptide sequence; hyperscore, the maximum X!Tandem hyperscore assigned to this peptide; srcFile, the name of the X!Tandem file from which the peptide assignment information was extracted; peptide, the peptide sequence; orf, the complete open-reading frame sequence that the peptide matches.

## Supplementary Material

Additional data file 1 The table gives the distribution of each type of diagnostic peptide among the 128 parent genes they occur in. The columns in the table are as follows. HUGO Gene ID, HUGO Gene Identifier; Ensembl Gene ID, the Ensembl gene identifier for the gene containing the diagnostic peptides; PT, the total number of diagnostic peptides found within the coding boundaries of this gene; PM, number of perfect-matching peptides to a protein product of this gene; IE, number of intra-exonic peptides associated with this gene; OE, number of exon overlapping peptides associated with this gene; NE, number of non-exonic peptides associated with this gene; Gene Description, a short descriptor characterizing the gene. The gene description is taken from the Ensembl genome browser's record for this gene Click here for file

Additional data file 2 The columns of the table are as follows: PT, the total number of NR peptides associated with this gene; All, the number of peptides with EST hits; PM, number of PM peptides with EST hits; IE, the number of IE peptides with EST hits; OE, the number of OE peptides with EST hits; NE, number of NE peptides with EST hits Click here for file

Additional data file 3 The table columns are as follows: Peptide Type, indicates whether the anchoring peptide is an NE or OE peptide; Ensembl Gene ID, reports the gene identifier for the gene the peptide occurs in; Chr, Start, and End, report the genomic nucleotide coordinates that encode for the peptide; ESTs, reports how many ESTs overlap with these genomic coordinates Click here for file

Additional data file 4 The columns in this spreadsheet are as follows: Ensembl Gene ID, the Ensembl gene identifier for the gene that the ORF overlaps; orflocid, a unique identifier for the ORF sequence; peplocid, a unique identifier for the peptide sequence identified as mapping to this ORF; groupId, an X!Tandem identifier for the spectrum assigned to the given peptide sequence; hyperscore, the maximum X!Tandem hyperscore assigned to this peptide; srcFile, the name of the X!Tandem file from which the peptide assignment information was extracted; peptide, the peptide sequence; orf, the complete open-reading frame sequence that the peptide matches Click here for file

## Figures and Tables

**Table 1:** Diagnostic peptides

	Number
**ORF identification statistics**	
All peptide matches above hyperscore 35	105,065
+ spectra match only a single peptide	66,711
+ peptide maps to a unique location in the genome	38,906
ORFs with confidence > 95%*	427
	
**Peptide classification**	
Intra-exonic perfect match (PM)	1,682
Intra-exonic different reading frame (IE)	47
Overlapping exon (OE)	90
Non-exonic (NE)	490

Peptides are categorized based upon where they align to in relation to the annotated start/stop boundaries of genes.*Based on Poisson statistic with correction for multiple hypothesis testing

**Table 2:** A representative set of peptide containing genes

HUGO gene ID	Ensembl gene ID	PT	PM	IE	OE	NE	Gene description
-	ENSG00000198209	28	26	0	2	0	Complement component 4B preproprotein
A1BG	ENSG00000121410	19	19	0	0	0	Alpha-1B-glycoprotein precursor (Alpha-1-B glycoprotein)
A2M	ENSG00000175899	47	46	0	0	1	Alpha-2-macroglobulin precursor (Alpha-2-M)
AFM	ENSG00000079557	12	11	0	0	1	Afamin precursor (Alpha-albumin; Alpha-Alb)
AGT	ENSG00000135744	21	21	0	0	0	Angiotensinogen precursor (contains angiotensin I (Ang I); angiotensin II (Ang II); angiotensin III (Ang III) (Des-Asp[1]-angiotensin II)).
AHSG	ENSG00000145192	24	24	0	0	0	Alpha-2-HS-glycoprotein precursor (Fetuin-A; Alpha-2-Z-globulin; Ba-alpha-2-glycoprotein)
ALB	ENSG00000163631	111	108	0	3	0	Serum albumin precursor
ANKRD24	ENSG00000089847	7	0	5	2	0	F20887_1, partial CDS (fragment)
APC2	ENSG00000115266	9	0	5	0	4	Adenomatosis polyposis coli 2
APCS	ENSG00000132703	11	11	0	0	0	Serum amyloid P-component precursor (SAP; 9.5S alpha-1-glycoprotein; contains serum amyloid P-component(1-203))
APOA1	ENSG00000118137	53	52	0	1	0	Apolipoprotein A-I precursor (Apo-AI; ApoA-I; contains apolipoprotein A-I(1-242))
APOA2	ENSG00000158874	17	15	0	2	0	Apolipoprotein A-II precursor (Apo-AII; ApoA-II; contains apolipoprotein A-II(1-76))
APOB	ENSG00000084674	112	110	0	2	0	Apolipoprotein B-100 precursor (Apo B-100; contains apolipoprotein B-48 (Apo B-48))
APOC3	ENSG00000110245	4	4	0	0	0	Apolipoprotein C-III precursor (Apo-CIII; ApoC-III)
APOE	ENSG00000130203	13	13	0	0	0	Apolipoprotein E precursor (Apo-E)
APOF	ENSG00000175336	4	4	0	0	0	Apolipoprotein F precursor (Apo-F)
APOH	ENSG00000091583	15	15	0	0	0	Beta-2-glycoprotein I precursor (apolipoprotein H; Apo-H; B2GPI; Beta(2)GPI; activated protein C-binding protein; APC inhibitor; anticardiolipin cofactor)
APOL1	ENSG00000100342	5	5	0	0	0	Apolipoprotein-L1 precursor (apolipoprotein L-I; apolipoprotein L; ApoL-I; Apo-L; ApoL)
AZGP1	ENSG00000160862	9	9	0	0	0	Zinc-alpha-2-glycoprotein precursor (Zn-alpha-2-glycoprotein; Zn-alpha-2-GP)
AZI1	ENSG00000141577	3	0	0	3	0	5-azacytidine induced 1 isoform a
BF	ENSG00000166285	9	7	0	2	0	Complement factor B precursor (EC 3.4.21.47; C3/C5 convertase; properdin factor B; glycine-rich beta glycoprotein; GBG; PBF2)

A breakdown of the distribution of diagnostic peptides among the 128 parent genes they occur in. HUGO gene ID, HUGO gene identifier; Ensembl gene ID, the Ensembl identifier for the gene containing the diagnostic peptides; PT, the total number of diagnostic peptides found within the coding boundaries of this gene; PM, number of perfect-matching peptides to a protein product of this gene; IE, number of intra-exonic peptides associated with this gene; OE, number of exon overlapping peptides associated with this gene; NE, number of non-exonic peptides associated with this gene; Gene description, the name given to the gene according to the Ensembl Genome Browser database. A complete list is available in Additional data file 1.

**Table 3:** EST library matches to diagnostic peptides

	PM	IE	NE	OE	Total
EST +	615 (72%)	24 (62%)	36 (17%)	36 (65%)	711
EST -	241 (28%)	15 (38%)	216 (83%)	19 (35%)	491
Total	856	39	252	55	1,202

A list of the breakdown of EST hits to a peptide in each of the four categories. EST +, indicates how many peptides in each category had at least one EST hit. EST -, gives the number of peptides in each category that did not match an EST. Percentages of total category total are given in parentheses. Totals are given in the final column and row. Only the longest representative peptide for a set of overlapping peptides was used in this analysis. PM, perfect matching peptide; IE, intra-exonic peptide; NE, non-exonic peptide; OE, overlapping exon peptide.

**Table 4:** Representative distribution of the ESTs across diagnostic peptides

HUGO gene ID	Ensembl gene ID	PT	ALL	PM	IE	OE	NE	Gene description
-	ENSG00000198209	17	17	15	0	2	0	Complement component 4B preproprotein
A1BG	ENSG00000121410	10	10	10	0	0	0	Alpha-1B-glycoprotein precursor (alpha-1-B glycoprotein)
A2M	ENSG00000175899	20	20	19	0	0	1	Alpha-2-macroglobulin precursor (alpha-2-M)
AFM	ENSG00000079557	4	3	3	0	0	0	Afamin precursor (alpha-albumin; alpha-Alb)
AGT	ENSG00000135744	13	13	13	0	0	0	Angiotensinogen precursor (contains angiotensin I (Ang I); angiotensin II (Ang II); angiotensin III (Ang III) (Des-Asp[1]-angiotensin II)).
AHSG	ENSG00000145192	9	9	9	0	0	0	Alpha-2-HS-glycoprotein precursor (fetuin-A; alpha-2-Z-globulin; Ba- alpha-2-glycoprotein)
ALB	ENSG00000163631	30	30	30	0	0	0	Serum albumin precursor
ANKRD24	ENSG00000089847	3	3	0	2	1	0	F20887_1, partial CDS (fragment)
APC2	ENSG00000115266	9	6	0	3	0	3	Adenomatosis polyposis coli 2
APCS	ENSG00000132703	7	7	7	0	0	0	Serum amyloid P-component precursor (SAP; 9.5S alpha-1-glycoprotein; contains serum amyloid P-component(1-203))
APOA1	ENSG00000118137	18	18	18	0	0	0	Apolipoprotein A-I precursor (Apo-AI; ApoA-I; contains apolipoprotein A-I(1-242))
APOA2	ENSG00000158874	5	5	4	0	1	0	Apolipoprotein A-II precursor (Apo-AII; ApoA-II; contains apolipoprotein A-II(1-76))
APOB	ENSG00000084674	95	95	94	0	1	0	Apolipoprotein B-100 precursor (Apo B-100; contains apolipoprotein B-48 (Apo B-48))
APOC3	ENSG00000110245	2	2	2	0	0	0	Apolipoprotein C-III precursor (Apo-CIII; ApoC-III)
APOE	ENSG00000130203	10	10	10	0	0	0	Apolipoprotein E precursor (Apo-E)
APOF	ENSG00000175336	4	4	4	0	0	0	Apolipoprotein F precursor (Apo-F)
APOH	ENSG00000091583	6	6	6	0	0	0	Beta-2-glycoprotein I precursor (apolipoprotein H; Apo-H; B2GPI; Beta(2)GPI; activated protein C-binding protein; APC inhibitor; anticardiolipin cofactor)
APOL1	ENSG00000100342	5	5	5	0	0	0	Apolipoprotein-L1 precursor (apolipoprotein L-I; apolipoprotein L; ApoL-I; Apo-L; ApoL)
AZGP1	ENSG00000160862	6	6	6	0	0	0	Zinc-alpha-2-glycoprotein precursor (Zn-alpha-2-glycoprotein; Zn- alpha-2-GP)
AZI1	ENSG00000141577	2	2	0	0	2	0	5-azacytidine induced 1 isoform a
BF	ENSG00000166285	5	5	4	0	1	0	Complement factor B precursor (EC 3.4.21.47; C3/C5 convertase; properdin factor B; glycine-rich beta glycoprotein; GBG; PBF2)

A representative sampling of the total number of ESTs matched to diagnostic peptides as well as the parent gene that contains the peptide. PT, total number of non-redundant (NR) peptides associated with this gene; All, number of peptides with EST hits; PM, number of PM peptides with EST hits; IE, number of IE with EST hits; OE, number of OE with EST hits; NE, number of NE with EST hits. A complete list is given in Additional data file 2.

**Table 5:** Features of proteins from genes with novel coding regions

HUGO gene ID	Ensembl gene ID	AAs in domain	Domain ID	Domain name	Gene name
PLG	ENSG00000122194	23	P00747	Kringle	Plasminogen precursor
BF	ENSG00000166285	28	P00751	Peptidase S1, trypsin	Complement factor B precursor
APOB	ENSG00000084674	21	Q13787	Vitellogenin	Apolipoprotein B-100 precursor
C4BPA	ENSG00000123838	29	P04003	Sushi	C4b-binding protein alpha chain precursor
HPX	ENSG00000110169	15	P02790	Hemopexin-like	Hemopexin precursor
GC	ENSG00000145321	17	P02774	Albumin	Vitamin D-binding protein precursor
PLEKHA4	ENSG00000105559	7	PS50003	PH_DOMAIN	Pleckstrin homology domain-containing protein family A member-4
IGLC1, IGLC2, IGLC3, IGLV1-40, IGLV3-25, IGLV4-3	ENSG00000100208	12	PS50835	IG-LIKE	Ig lambda chain C region
IGHA1, IGHG3, IGHM	ENSG00000130076	11	PS50835	IG-LIKE	Ig alpha-1 chain C region
-	ENSG00000142082	51	PS50305	SIRTUIN	NAD-dependent deacetylase sirtuin-3 mitochondrial precursor
TF	ENSG00000091513	11	PS00207	TRANSFERRIN	Serotransferrin precursor

A list of the protein domains that the novel OE peptides overlapped. HUGO gene ID, Hugo gene identifiers; Ensembl gene ID, Ensembl gene identifier; AAs in domain, number of amino acids from the peptide that are part of the domain; Domain ID, the Uniprot or Prosite identifier for the domain (Prosite identifiers begin with the letters 'S'); Domain name, the common name assigned to the domain in either Uniprot or Prosite.
